# Prognostic Value of FOXM1 in Patients with Malignant Solid Tumor: A Meta-Analysis and System Review

**DOI:** 10.1155/2015/352478

**Published:** 2015-07-22

**Authors:** Jun Dai, Lili Yang, Jinyu Wang, Ying Xiao, Qiurong Ruan

**Affiliations:** ^1^Institute of Pathology, Tongji Hospital of Tongji Medical College, Huazhong University of Science and Technology, Wuhan 430030, China; ^2^Department of Pediatrics, The Second Affiliated Hospital, Zhejiang University College of Medicine, Hangzhou 310009, China

## Abstract

Forkhead box M1 (FOXM1), a member of the Fox transcription factors family, was closely related with cell cycle. FOXM1 played an important role in MST and prompted a poor prognosis for MST patients. However, there were also some studies revealing no significant association between the FOXM1 expression and prognosis of patients. Therefore, we conducted meta-analysis to investigate whether the expression of FOXM1 was associated with MST prognosis. We collected 36 relevant studies through PubMed database and obtained research data of 4946 patients. Stata 12.0 was used to express the results as hazard ratio (HR) for time-to-event outcomes with 95% confidence intervals (95% CI). It was shown that overexpression of FOXM1 was relevant to worse survival of MST patients (HR = 1.99, 95% CI = 1.79–2.21, *P* < 0.001; *I* ^2^ = 26.4%, *P*_*h*_ = 0.076). Subgroup analysis suggested that overexpression of FOXM1 in breast cancer (BC), gastric cancer (GC), hepatocellular carcinoma (HCC), pancreatic ductal adenocarcinoma (PDA), and non-small-cell lung cancer (NSCLC) all predicted a worse survival (*P* < 0.05), in addition to ovarian cancer (OC) (*P* = 0.084). In conclusion, our research indicated that overexpression of FOXM1 was to the disadvantage of the prognosis for majority of MST and therefore can be used as an evaluation index of prognosis.

## 1. Introduction

MST collectively referred to a large class of diseases which has a wide variety. Its morbidity and mortality vary with tumor types; however, the death toll ranks the first in the world [1].

With gradual deeper understanding of MST, molecular targeted therapy has become a major means [2, 3]. FOXM1 is an important member of the Forkhead transcription factors family. Microarray study confirmed that the expression of FOXM1 increased in most solid tumors [4]. FOXM1, which plays key roles in cell evolution from G1 to S phase and the maintenance of chromosome stability, is an important transcription factor to regulate the proliferation and apoptosis of cells [5–7]. FOXM1 can not only promote the formation of tumor by increasing cell proliferation ability, but also enhance tumor metastasis and invasion activity in advanced cancer [8–10]. Therefore, it may be said that FOXM1 fully participates in the development and progression of tumors. Numerous studies reported that FOXM1 had predictive value for MST prognosis, including BC, NSCLC, HCC, GC, and cervical cancer, [11–15]. However, there were also some studies revealing no significant association between FOXM1 and the prognosis of MST patients [16].

As factors affecting prognosis were numerous and miscellaneous, we can not simply evaluate whether one study was representative. Therefore, we conducted meta-analysis of the relationship between FOXM1 expression and prognosis in MST patients, expecting to eliminate or weaken the deficiency in studies through expanding the sample size and thereby restore the real predictive value of FOXM1.

## 2. Materials and Methods

### 2.1. Document Retrieval

We collected studies that have been published before January 2015 through PubMed using the following terms: (“cancer” or “tumor” or “tumour” or “neoplasm”) and (“FOXM1” or “FOXM1a” or “FOXM1b” or “FOXM1c”). We first excluded apparently unrelated studies by browsing the titles and abstracts. The full texts of all potentially eligible studies were retrieved, and their references were carefully browsed to find other studies that met the criteria. Search eligible studies by two authors independently and finally negotiate to reach consensus. The criteria were as follows: (I) research on the relationship between FOXM1 expression and the prognosis of patients in MST. (II) In the original data provided, FOXM1 expression must be divided into two grades: “positive” and “negative,” regardless of FOXM1 mRNA or protein detection and the detection methods. (III) Studies must provide available data, such as HR and 95% CI, survival curve. Similarly, we also drew up a number of exclusion criteria: (I) studies belonging to case reports, reviews, or meta-analysis, (II) authors providing only odds ratio (OR) or relative risk (RR) (we can not directly or indirectly obtain information such as HR and 95% CI), and (III) research on leukemia.

### 2.2. Qualitative Assessment

In order to better assess the quality of the qualified studies, two independent researchers drew up the evaluation program for this study and evaluated the studies included. In short, the scoring method was based on the following five aspects: whether the diagnosis of cancer was clear; numbers of the cases; representativeness of the cases collected; judgment criteria of FOXM1; and the data sources of HR (95% CI). Each item got the maximum score of 2 points and the minimum score of 1 point (see Supplemental Table  1 in Supplementary Material available online at http://dx.doi.org/10.1155/2015/352478). The total points ranged from 5 to 10 points. Points greater than or equal to 9 points were considered to be high-quality researches.

### 2.3. Data Extraction

Two authors extracted information independently according to predetermined extract forms. Extract data from eligible studies included first author, year, regions, numbers of patients, tumor types, determination method of FOXM1, percentage of FOXM1 overexpression, and the sources of HR (95% CI). If data in any of the above categories had not been reported in the original document, items were deemed as “Not Report.” If the results of univariate and multivariate analysis were both reported in a study, the latter was chosen for the reason that it took the confounding factor into account and therefore it was more accurate. Taking into account the number and time of the events, the HR was the most appropriate statistic for the follow-up assessment to analyze time-to-event outcomes. For each study, the HR (95% CI) were evaluated according to the data provided in the articles. If accurate HR (95% CI) were given in the study, we can directly use them. In other cases, we can use Engauge Digitizer version 2.11 software to extract relevant numerical value from survival curves and calculate the HR (95% CI) while only Kaplan-Meier survival curves were provided in the original texts [17, 18].

### 2.4. Statistical Analysis

Cochran's *Q* and *I* ^2^ statistics were used to calculate the heterogeneity of the individual HR. As to *Q* statistic, *P* < 0.05 was considered to have statistical significance. For *I* ^2^ statistics, *I* ^2^ < 25%, no heterogeneity; *I* ^2^ = 25–50%, moderate heterogeneity; and *I* ^2^ > 50%, strong heterogeneity [19, 20]. The fixed effects model was used to combine the individual HR estimates while no significant heterogeneity was found among studies, or else, the random effects model was applied. For FOXM1 overexpression groups, HR > “1” indicated worse survival. If there was no overlap between 95% CI and “1,” it would be considered that the impact of FOXM1 on survival was statistically significant. Publication bias was tested through Begg's test and Egger's test; *P* < 0.05 was considered to be statistically significant. STATA 12.0 software (Stata Corporation, College Station, TX) was used to conduct all statistical analyses.

## 3. Results

### 3.1. Characteristics of Eligible Studies

**Figure 1:**
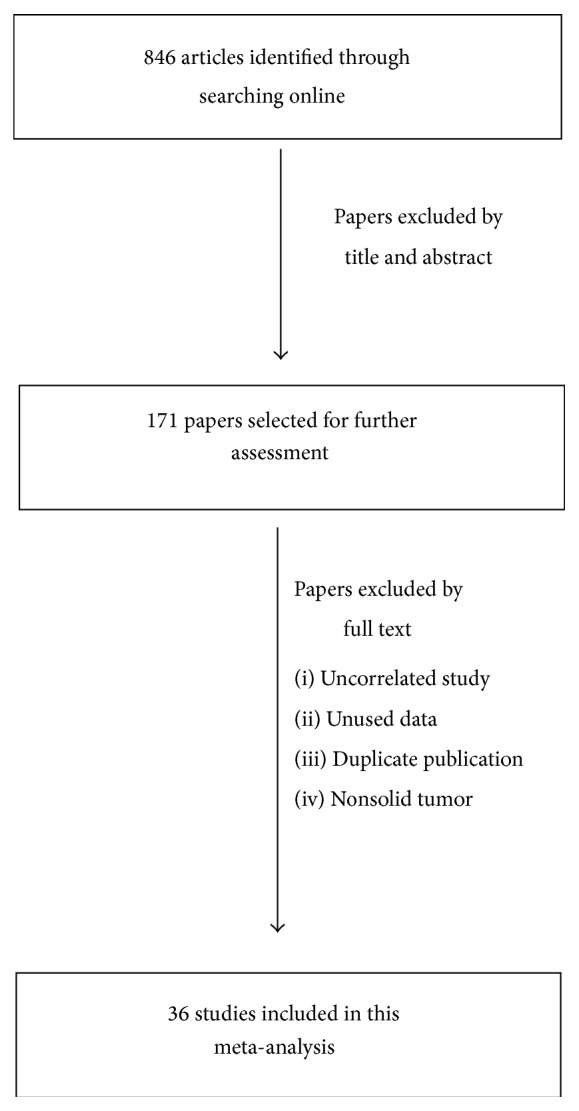
Flowchart of study selection in this meta-analysis. 846 studies were preretrieved in accordance with the established search strategies. Then 171 studies that may meet the requirements were further screened out through browsing the titles and abstracts. After reading the full texts of 171 studies, 36 eligible studies were finally included in this meta-analysis according to the criteria.

846 studies were preretrieved in accordance with the established search strategies. Then 171 studies that may meet the requirements were further screened out through browsing the titles and abstracts. After reading the full texts of 171 studies, 36 eligible studies were finally included in this meta-analysis according to the criteria (Figure 1). For studies included, the tumor types contained BC [11, 16, 21, 22], cervical cancer [12], colorectal cancer [23, 24], esophageal cancer [25], gallbladder cancer [26], GC [14, 27, 28], glioblastoma [29], HCC [13, 30–32], laryngeal cancer [33], NSCLC [15, 34–39], malignant peripheral nerve sheath tumor (MPNST) [40], medulloblastoma [41], OC [42–44], PDA [45–47], clear cell renal cell carcinoma (CCRCC) [48, 49], and oral cancer [35]. Characteristics of eligible studies were summarized in Table 1. Eligible studies included 4946 patients and the number of samples per study ranged from 38 to 455, with an average of 137. Eligible studies were mainly distributed in Asia (31 studies), while only 5 studies were found in non-Asia. Immunohistochemistry (IHC) was used to detect the expression of FOXM1 in vast majority of the studies. Except that one study did not mention the percentage of FOXM1 overexpression, the lowest percentage of FOXM1 overexpression was 20%, while the highest was 78.7%, with an average of 57.36%. Among 36 studies, 16 studies provided available HR and 95% CI, whereas 20 studies only provided survival curves. According to the qualitative assessment criteria, there were 21 studies with total scores greater than or equal to 9 points, while there was no study with less than 7 points.

### 3.2. Meta-Analysis

**Figure 2:**
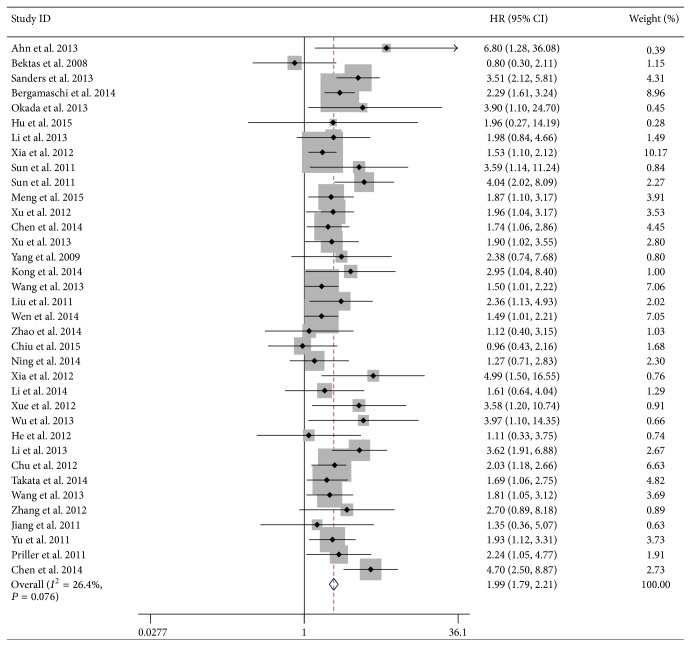
Meta-analysis of the association between FOXM1 expression and survival in MST. Combine the data of 36 studies on MST to assess the survival. The results indicated statistically significant difference (HR = 1.99, 95% CI = 1.79–2.21, and *P* < 0.001) and slight between-research heterogeneity (*I* ^2^ = 26.4%, *P*_*h*_ = 0.076) between MST prognosis and FOXM1 expression.

All the main results of this meta-analysis were shown in Table 2. Combine the data of 36 studies on MST to assess the cumulative survival (Figure 2). The results indicated statistically significant difference (HR = 1.99, 95% CI = 1.79–2.21, and *P* < 0.001) and slight between-research heterogeneity (*I* ^2^ = 26.4%, *P*_*h*_ = 0.076) between MST prognosis and FOXM1 expression.

We conducted subgroup analysis based on information provided by these studies (Table 2). We found that FOXM1 overexpression had a statistically significant effect on survival in both Asian groups and non-Asian groups, with the HR of 1.92 (95% CI = 1.71–2.16) and 2.28 (95% CI = 1.81–2.89), respectively. In Asian groups, no heterogeneity was found among studies (*I* ^2^ = 21.6%, *P*_*h*_ = 0.143); however, there was a slight heterogeneity in non-Asian groups (*I* ^2^ = 47.4%, *P*_*h*_ = 0.107). With 100 patients as the boundary, these two groups all prompted that FOXM1 overexpression was not conducive to prognosis (*P* < 0.001), and there was no significant heterogeneity (*I* ^2^ < 50%). Moreover, we performed grouping according to the data source of HR (95% CI) and scores derived from the rating scale we have established, respectively. In both cases, all groups were statistically significant (*P* < 0.001), and no obvious heterogeneity existed among them (*I* ^2^ < 50%).

Finally, we divided tumors from the same organ into one group. Four studies related to BC were divided into the same group, with the combined HR = 2.46 (95% CI = 1.87–3.22). There was significant statistical significance between the expression of FOXM1 and the prognosis of BC (*P* < 0.001). However, the heterogeneity among various studies was very high (*I* ^2^ = 65.1%, *P*_*h*_ = 0.035). Similar situation occurred in HCC group, with the combined HR = 1.90 (95% CI = 1.48–2.44, *P* < 0.001; *I* ^2^ = 59.5%, *P*_*h*_ = 0.06). In GC group, the combined HR = 2.27 (95% CI = 1.13–4.58, *P* = 0.022; *I* ^2^ = 0%, *P*_*h*_ = 0.747). In PDA group, the combined HR = 1.73 (95% CI = 1.05–2.86, *P* = 0.032; *I* ^2^ = 47.2%, *P*_*h*_ = 0.151). There were 7 studies in NSCLC group, the combined HR = 1.82 (95% CI = 1.46–2.28; *I* ^2^ = 0%, *P*_*h*_ = 0.864), which showed a significant statistical significance (*P* < 0.001) and no heterogeneity. Surprisingly, the 95% CI of HR in ovarian cancer showed an overlap with 1 (*P* = 0.084). In addition, oral cancer, laryngeal SCC, cervical cancer, and esophagus cancer among 36 studies that were included were all identified as SCC. Individual analysis of these studies revealed that FOXM1 also had predictive value in SCC (HR = 2.20, 95% CI = 1.55–3.12, *P* < 0.001; *I* ^2^ = 64.4%, *P*_*h*_ = 0.038).

### 3.3. Publication Bias

**Figure 3:**
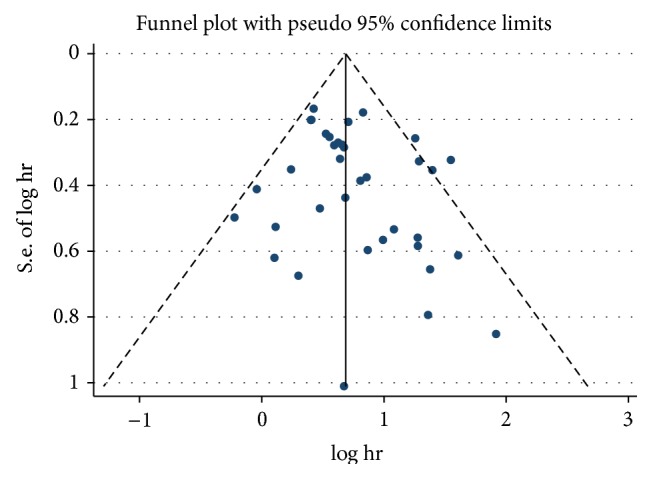
Funnel plots for study of MST patients. Funnel plot analysis showed no publication bias among the 36 studies included.

**Figure 4:**
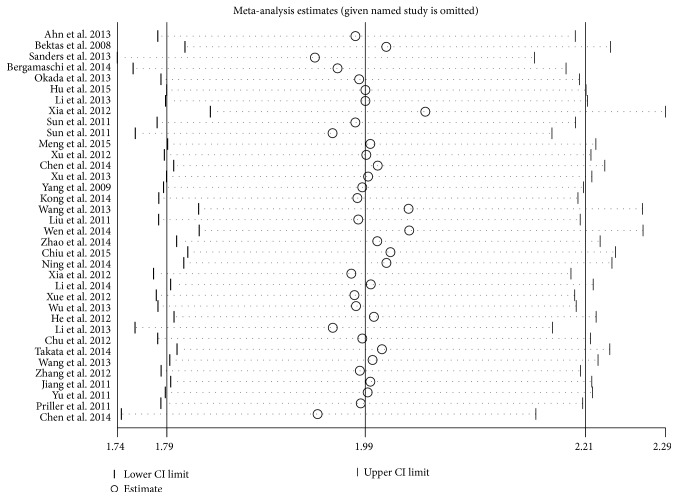
Sensitivity analysis. The sensitivity analysis showed that no individual study significantly influenced the combined HR, indicating the robust result of this meta-analysis.

Funnel plot analysis showed no publication bias among the 36 studies included (Figure 3). In order to avoid influence of subjective judgment on the conclusion, we validated whether there was publication bias using Begg's test and Egger's test. Indeed, the results revealed no evidence of publication bias (*P*_begg_ = 0.089, *P*_egger_ = 0.127). Furthermore, we also expressed the results by the fixed effect model so as to compare the differences and evaluate the sensitivity of the meta-analyses. The sensitivity analysis showed that no individual study significantly influenced the combined HR, indicating the robust result of this meta-analysis (Figure 4).

## 4. Discussion

Information provided by individual study was limited. To our knowledge, this meta-analysis was the first study to systematically assess the association between FOXM1 expression and the prognostic factors of MST. Our meta-analysis and systematic review showed that the prognosis of patients with tumors overexpressing FOXM1 was significantly worse than patients with low expression of FOXM1.

How FOXM1 affected the prognosis of patients and what role FOXM1 played in MST have gradually become clear. FOXM1 had the common characteristic of Fox transcription factors family: a conserved DNA sequence with winged helix domain [50]. Human FOXM1 gene, which was about 25 kb, was located in 12p13-3 chromosome band (telomere position) and composed of ten exons [51]. FOXM1 was involved in the development and growth of the tumors. The most important function of FOXM1 was to regulate cell cycle, promote cell proliferation, and inhibit cell aging and apoptosis. Cell cycle and its functioning were an orderly process of gene regulation, with the participation of a number of cyclins and cyclin-dependent kinases (Cdks). In quiescent cells, FOXM1 was hardly expressed, while, in cell proliferation, FOXM1 showed high expression. It was involved in the transcription regulation of many genes related to cell cycle and thereby controlled the cellular DNA replication and mitosis process [52, 53]. Pathological overexpression of FOXM1 can induce the malignant proliferation of tumor cells. The main signaling pathways involved included FOXM1-Skp2-p27 [54, 55], FOXM1-ras-ROS [56, 57], and FOXM1-Raf/MEK/MAPK [58]. In addition, FOXM1 can activate the transcription of C-myc, which was an important signaling molecule to stimulate cell proliferation [59, 60]. On the other hand, FOXM1 also regulated the expression of Cdc25A phosphatase that was necessary for activating DNA replication; therefore, FOXM1 missing can naturally inhibit mitosis process [53]. FOXM1 was closely related to cell senescence as well. FOXM1 suppressed the expression of p53 and thereby significantly inhibited cellular aging [61, 62]. In short, FOXM1 maintained the homeostasis between cell proliferation and apoptosis; breaking the balance may promote tumorigenesis [4]. In addition, FOXM1 was also involved in the invasion and metastasis of tumors. It has shown that FOXM1 promoted tumor metastasis by regulating epithelial-mesenchymal conversion (EMT) in tumor cells [63, 64]. However, some studies demonstrated that FOXM1 promoted tumor metastasis by regulating the expression of MMPs [13, 65–67]. Moreover, FOXM1 also accelerated angiogenesis via upregulation of VEGF expression and thereby promoted tumor metastasis [68, 69]. All in all, these results suggested that FOXM1 promoted or facilitated the invasion and metastasis of tumor cells through a variety of pathways. In summary, FOXM1 was involved in tumor proliferation, invasion, metastasis, and angiogenesis by regulating the expression of downstream genes related to the tumor and thereby affected the prognosis of patients.

Our research revealed that FOXM1 played a potentially important role in MST; however, we should be extra cautious when applying these results into clinical practice. As many types of tumors were contained in 36 included studies, the biological characteristics of the tumors were various. To further investigate the predictive value of FOXM1 in different types of tumors, we analyzed the combined HR of different groups. In BC group, we obtained meaningful results after combining the HR of 4 studies; however, the heterogeneity was great (*I* ^2^ > 50%). Among these 4 studies, one study was targeted for breast cancer of all types, while the other three opted for ER-positive BC. After excluding this study, we got more accredited results (HR = 2.70, 95% CI = 2.04–3.59, *P* < 0.001; *I* ^2^ = 34.8%, *P*_*h*_ = 0.216). As a consequence, we were more inclined to believe that FOXM1 had a reliable predictive significance in ER-positive BC patients; nonetheless, this did not mean that FOXM1 did not have predictive value in BC of all types. More study will be able to make up for the vacancy in the future. In GC, PDA, and HCC group, overexpression of FOXM1 all prompted worse prognosis in patients. In GC group, 95% CI overlapped with “1” in some studies [27, 28]. Likewise, in PDA group, 95% CI in some studies also overlapped with “1” [45, 47]. Even when there were only 3 studies combined, the results were still meaningful. In HCC group, although it had significant statistical significance, its heterogeneity was very obvious. In NSCLC group, 7 studies were included. The conclusion that overexpression of FOXM1 prompted worse prognosis in patients also applied to NSCLC, but it was more stable and reliable (*I* ^2^ = 0%).

Publication bias was a problem to be analyzed in all of the meta-analysis. In this study, we found no publication bias by Begg's test and Egger's test. Funnel plot also revealed no obvious bias. However, some studies had not been included because they did not meet our screening conditions. If we can get the raw data from these studies, reanalysis will be needed. In addition, although analyzing the time-to-event outcomes via the HR had very distinct advantages, there was also a problem. Since follow-up time of each study was not the same, heterogeneity was virtually brought in. Due to the limitation of statistical methods, we should be particularly cautious in the interpretation of the results.

In conclusion, our research suggested that overexpression of FOXM1 may lead to poor prognosis. With the gradual deepening of the study, FOXM1 may become an important target for cancer therapy in the future.

## Supplementary Material

Supplementary Material: Qualitative assessment of the qualified studies. In order to better assess the quality of the qualified studies, two independent researchers drew up the evaluation program for this study and evaluated the studies included.

## Figures and Tables

**Table 1:** Characteristics of eligible studies.

Study	Country	Cancer type	Duration	Number	Method	Percent	Follow-up assessment		Quality
Bektas et al. 2008 [16]	Germany	BC	1994–2002	204	IHC	72.3%	SC	0.80 (0.30–2.11)	9
Sanders et al. 2013 [11]	UK	BC; ER+	NR	455	Microarray	49.9%	HR	3.51 (2.12–5.81)	9
Bergamaschi et al. 2014 [21]	USA	BC; ER+	NR	501	IHC	20.2%	SC	2.29 (1.61–3.24)	8
Ahn et al. 2013 [22]	Korea	BC; ER+	1997–2007	81	Microarray	29.6%	SC	6.80 (1.28–36.08)	8
He et al. 2012 [12]	China	Cervical cancer; early-stage	2002–2005	102	IHC	73.5%	SC	1.11 (0.33–3.75)	9
Li et al. 2013 [23]	China	Colon cancer;	2001–2003	203	IHC	75.8%	HR	3.62 (1.91–6.88)	10
Chu et al. 2012 [24]	China	Colorectal cancer	2002–2004	112	IHC	50.9%	HR	2.03 (1.18–2.66)	10
Takata et al. 2014 [25]	Japan	Esophageal cancer	2001–2007	174	IHC	50%	HR	1.69 (1.06–2.75)	10
Wang et al. 2013 [26]	China	Gallbladder cancer	2002–2007	76	IHC	56.6%	SC	1.81 (1.05–3.12)	9
Okada et al. 2013 [14]	Japan	GC	2001–2008	77	IHC	68.8%	HR	3.9 (1.1–24.7)	9
Hu et al. 2015 [27]	China	GC	NR	40	IHC	65%	SC	1.96 (0.27–14.19)	7
Li et al. 2013 [28]	China	GC	2007	103	IHC	78.6%	SC	1.98 (0.84–4.66)	9
Zhang et al. 2012 [29]	China	Glioblastoma	NR	38	IHC	71.1%	SC	2.70 (0.89–8.18)	7
Xia et al. 2012 [13]	China	HCC	1999–2001	306	IHC	65.7%	HR	1.53 (1.10–2.12)	10
Sun et al. 2011 [30]	China	HCC	2001–2009	99	IHC	42.4%	SC	3.59 (1.14–11.24)	8
Sun et al. 2011 [31]	China	HCC	2001–2008	150	IHC	59.3%	SC	4.04 (2.02–8.09)	9
Meng et al. 2015 [32]	China	HCC	2006–2010	172	IHC	62.2%	SC	1.87 (1.10–3.17)	9
Jiang et al. 2011 [33]	China	Laryngeal SCC	2002–2003	89	IHC	78.7%	SC	1.35 (0.36–5.07)	8
Xu et al. 2012 [34]	China	NSCLC	2005–2008	201	IHC	NR	HR	1.96 (1.04–3.17)	10
Chen et al. 2014 [35]	Taiwan	NSCLC	1993–2004	117	IHC	60.7%	HR	1.74 (1.06–2.86)	10
Xu et al. 2013 [15]	China	NSCLC	2005–2008	175	IHC	64%	HR	1.899 (1.016–3.551)	10
Yang et al. 2009 [36]	Korea	NSCLC	2000–2004	69	IHC	37.7%	SC	2.38 (0.74–7.68)	8
Kong et al. 2014 [37]	China	NSCLC	2004–2006	68	IHC	63.2%	HR	2.95 (1.036–8.397)	9
Wang et al. 2013 [38]	China	NSCLC; stage IIIb-IV	2004–2007	162	IHC	71.6%	SC	1.50 (1.01–2.22)	9
Liu et al. 2011 [39]	China	NSCLC	2004–2008	68	IHC	36.8%	SC	2.36 (1.13–4.93)	8
Yu et al. 2011 [40]	USA	MPNST	NR	82	IHC	70.8%	HR	1.93 (1.12–3.31)	8
Priller et al. 2011 [41]	Germany	Medulloblastoma	NR	130	IHC	33.8%	HR	2.24 (1.05–4.77)	9
Chen et al. 2014 [35]	Taiwan	Oral cancer	1993–2004	110	IHC	43.6%	HR	4.70 (2.50–8.87)	10
Wen et al. 2014 [42]	China	OC	2009–2011	158	IHC	63.9%	HR	1.494 (1.007–2.215)	10
Zhao et al. 2014 [43]	China	OC	1987–2004	82	IHC	64.6%	SC	1.12 (0.40–3.15)	8
Chiu et al. 2015 [44]	Taiwan	OC	1993–2010	106	IHC	44.3%	SC	0.96 (0.43–2.16)	9
Ning et al. 2014 [45]	China	PDA; stage II	2002–2013	136	IHC	63.2%	HR	1.270 (0.713–2.830)	10
Xia et al. 2012 [46]	China	PDA	2003–2007	80	IHC	66.3%	SC	4.99 (1.50–16.55)	8
Li et al. 2014 [47]	China	PDA	NR	50	IHC	56%	SC	1.61 (0.64–4.04)	7
Xue et al. 2012 [48]	China	CCRCC	2004–2008	83	IHC	45.8%	SC	3.58 (1.20–10.74)	8
Wu et al. 2013 [49]	China	CCRCC	2006–2010	87	IHC	42.5%	HR	3.97 (1.10–14.35)	9

BC: breast cancer; GC: gastric cancer; HCC: hepatocellular carcinoma; SCC: squamous cell carcinoma; NSCLC: non-small-cell lung cancer; MPNST: malignant peripheral nerve sheath tumor; OC: ovarian cancer; PDA: pancreatic ductal adenocarcinoma; CCRCC: clear cell renal cell carcinoma; NR: Not Report; IHC: immunohistochemistry; HR: hazard ratio; SC: survival curve; ER(+): Estrogen Receptor (+).

**Table 2:** Meta-analysis.

	Study	HR (95% CI)	*P*	*I* ^2^	*P*_*h*_
Region					
Asian	31	1.92 (1.71–2.16)	<0.001	21.6%	0.143
Non-Asian	5	2.28 (1.81–2.89)	<0.001	47.4%	0.107
Number of patients					
≤100	16	2.27 (1.79–2.88)	<0.001	0%	0.814
>100	20	1.93 (1.71–2.16)	<0.001	47.2%	0.011
Sources of HR					
HR	16	2.01 (1.75–2.30)	<0.001	39.2%	0.055
SC	20	1.96 (1.67–2.31)	<0.001	17%	0.242
Quality score					
5–8	13	2.27 (1.82–2.83)	<0.001	0%	0.785
9-10	23	1.91 (1.70–2.15)	<0.001	41.7%	0.020
Cancer type					
BC	4	2.46 (1.87–3.22)	<0.001	65.1%	0.035
GC	3	2.27 (1.13–4.58)	0.022	0%	0.747
HCC	4	1.90 (1.48–2.44)	<0.001	59.5%	0.060
NSCLC	7	1.82 (1.46–2.28)	<0.001	0%	0.864
PDA	3	1.73 (1.05–2.86)	0.032	47.2%	0.151
OC	3	1.34 (0.96–1.88)	0.084	0%	0.587
Others	12	2.04 (1.72–2.42)	<0.001	27.6%	0.159
Overall	36	2.02 (1.81–2.25)	<0.001	25.6%	0.09
