# Supplementary material for: Prognostic Value of FOXM1 in Patients with Malignant Solid Tumor: A Meta-Analysis and System Review
Source: Dis Markers. 2015 Jul 22;2015:352478. doi: 10.1155/2015/352478 (PMC4584221; doi:10.1155/2015/352478)
Supplement: Supplementary file 1 — Supplementary Material: Qualitative assessment of the qualified studies. In order to better assess the quality of the qualified studies, two independent researchers drew up the evaluation program for this study and evaluated the studies included. [file 352478.f1.doc]

**Supplemental Table 1. Qualitative assessment**

| criteria |  | Score |
| --- | --- | --- |
| diagnosis | Clear | 2 |
|  | Unclear | 1 |
| numbers of patients | >100 | 2 |
|  | <100 | 1 |
| Consecutive patients | Yes | 2 |
|  | Unclear | 1 |
| FOXM1 judgment | Detailed criteria | 2 |
|  | No description | 1 |
| Data source | HR and 95%CI | 2 |
|  | Survival curve | 1 |
